# Internalization of affinity tags enables the purification of secreted Chlamydomonas proteins

**DOI:** 10.1007/s00294-025-01311-2

**Published:** 2025-03-19

**Authors:** Anna Probst, Doreen Knochenhauer, Justus Niemeyer, Laura Fischer, Michael Schroda

**Affiliations:** Molecular Biotechnology & Systems Biology, RPTU Kaiserslautern-Landau, Paul- Ehrlich-Straße 23, D-67663 Kaiserslautern, Germany

**Keywords:** Synthetic biology, 2S albumin, SARS-CoV-2 spike protein, Protein secretion, Affinity purification, Golden gate cloning, Microalgae

## Abstract

There is great interest in establishing microalgae as new platforms for the sustainable production of high-value products such as recombinant proteins. Many human therapeutic proteins must be glycosylated, which requires their passage through the secretory pathway into the culture medium. While the low complexity of proteins in the culture medium should facilitate affinity purification of secreted recombinant proteins, this has proven challenging for proteins secreted by the unicellular green alga *Chlamydomonas reinhardtii*. In *Leishmania tarentulae*, we observed that C-terminally exposed affinity tags are frequently truncated, presumably due to proteolytic activity. We wondered whether this might also occur in Chlamydomonas and contribute to the difficulties in affinity purification of secreted proteins in this alga. Using the methionine-rich 2S albumin from *Bertholletia excelsa* and the ectodomain of the SARS-CoV-2 spike protein produced and secreted in Chlamydomonas, we demonstrate that they can be efficiently affinity-purified from the culture medium by Ni-NTA chromatography when the 8xHis affinity tag is internalized. This finding represents an important step towards further development of Chlamydomonas as a host for the sustainable production of high-value recombinant proteins.

**Supplementary Information:**

The online version contains supplementary material available at 10.1007/s00294-025-01311-2.

## Introduction

Microalgae are considered promising sustainable hosts for biotechnology, as they grow rapidly, rely on light and simple mineral media, efficiently capture CO_2_ and have a high land use efficiency, without competing with arable land (Einhaus et al. 2024). The hype about using microalgae for the production of biofuels was followed by disillusionment as the cultivation and harvesting costs remain higher than the value of the biofuels produced (Sarwer et al. 2022). To improve the ability of microalgae to produce biofuels cost-efficiently, genetic modifications appear to be essential, though their implementation may take many years. To set the stage for this it might be a good strategy to first use microalgae as hosts for the production of high-value products. This would facilitate the development of appropriate molecular tools while simultaneously generating profit. To date, *Chlamydomonas reinhardtii* (Chlamydomonas) is the most developed microalgal system for genetic engineering with robust protocols for gene editing via CRISPR/Cas9 (Chen et al. 2023; Ferenczi et al. 2017), dedicated expression strains (Neupert et al. 2009), a Golden-Gate-based library of genetic parts enabling the rapid assembly of multi-gene constructs within a few days (Crozet et al. 2018), and simple transgene delivery methods (Kindle 1990; Shimogawara et al. 1998). Importantly, our knowledge of how to design nuclear transgenes for robust expression in Chlamydomonas has grown. This includes optimizing codon usage (Barahimipour et al. 2015), regularly interrupting the coding sequence with introns (Baier et al. 2018b), and using strong promoters in combination with suitable 5’- and 3’-UTRs (Fischer and Rochaix 2001; Lopez-Paz et al. 2017; Niemeyer et al. 2023; Schroda et al. 2000). This knowledge of nuclear transgene design has been used successfully for the production of high-value compounds such as sesquiterpenoids (Gutiérrez et al. 2024), astaxanthin (Amendola et al. 2023), ε-caprolactone (Siitonen et al. 2023), putrescine (Freudenberg et al. 2022), or cadaverine (Freudenberg et al. 2021). Even human therapeutic proteins have been produced in Chlamydomonas, including the SARS-CoV-2 receptor-binding domain and full ectodomain (Berndt et al. 2021; Kiefer et al. 2022), intercellular adhesion molecule 1 (Torres-Tiji et al. 2022), interleukin-2 (Dehghani et al. 2020), human pro-angiogenic growth factors (Chavez et al. 2016; Jarquin-Cordero et al. 2020), human epidermal growth factor (Baier et al. 2018a), and erythropoietin (Eichler-Stahlberg et al. 2009). Since many human therapeutic proteins must be glycosylated for being active, their targeting to the secretory pathway is essential. The secreted proteins accumulate in the culture medium, where the lower complexity of proteins should facilitate purification of recombinant proteins. While there are reports of successful purification of recombinant proteins with C-terminal poly-histidine tags from Chlamydomonas culture medium (Eichler-Stahlberg et al. 2009; Lauersen et al. 2013; Perozeni et al. 2023), we have not been able to purify the secreted SARS-CoV-2 ectodomain with a C-terminal octa-histidine tag (Kiefer et al. 2022). Since, in the studies reporting successful affinity purification, the recombinant proteins were only detected by immunoblotting, the purification appears to have been rather inefficient. We found that C-terminal tags on recombinant proteins produced and secreted in *Leishmania tarentula* are cleaved from a large fraction of these proteins, presumably by peptidases and proteases (Hieronimus et al. 2024). We wondered whether this problem also exists in Chlamydomonas and whether it could be overcome by the internalization of the affinity tag.

## Results

**Fig. 1:**
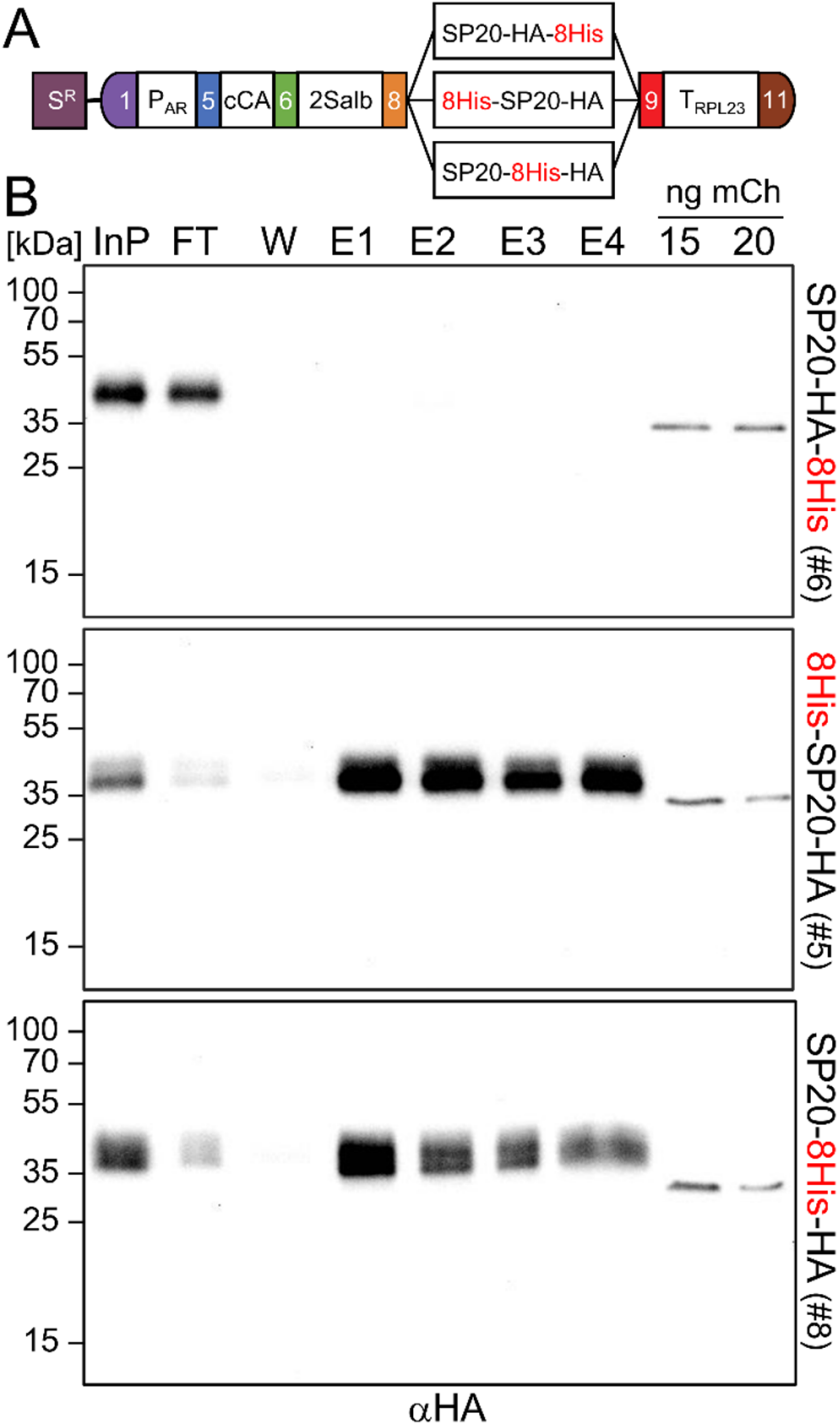
Ni-NTA purification of secreted 2S albumin harboring the 8xHis tag at different positions. **A** Level 2 constructs for the production and secretion of 2S albumin in Chlamydomonas with the 8xHis tag at different positions. P_AR_– *HSP70A-RBCS2* promoter; cCA– secretion signal from carbonic anhydrase; 2Salb– coding sequence for 2S albumin; SP20– glycomodule of 20 serine-proline repeats; HA– hemagglutinine epitope; 8xHis– 8x histidine affinity tag; T_RPL23_– 3'-UTR of the *RPL23* gene. The *aadA* cassette under control of the *PSAD* promoter and terminator (S^R^) was used as selection marker. **B** Purification of 2S albumin from the culture medium of transformants expressing the constructs depicted in A). 2S albumin was purified from 100 ml cell-free culture medium from transformants #6, #5, and #8 (Supplemental Fig. 1) via Ni-NTA affinity chromatography. Proteins in the indicated fractions (1 ml each) were precipitated with TCA and analyzed by immunoblotting. Recombinant mCherry-6His-1HA (mCh) from Kiefer et al. (2022) was loaded as a positive control for the antibody and to allow for comparing signals between blots. InP– input; FT– flow-through; W– wash; E1-4– elution fractions with 500 mM imidazol. Shown are representative replicates from three experiments (all replicates are shown in Supplemental Fig. 2)

We wanted to test whether the problem of purification of secreted proteins with C-terminal affinity tags from Chlamydomonas culture medium could be due to the removal of the exposed tags. To this end, we generated three genetic parts for the B5 position according to the Chlamydomonas Modular Cloning standard (Crozet et al. 2018) encoding C-terminal fusions with different positions of the 8xHis affinity tag (Fig. 1A). All parts also contain the synthetic SP20 module consisting of 20 serine/proline repeats shown to enhance the yield of secreted proteins in Chlamydomonas severalfold (Ramos-Martinez et al. 2017) as well as the HA epitope for immunodetection. As a test protein we used the methionine-rich 2S albumin from *Bertholletia excelsa*. For high-level expression, the coding sequence for 2S albumin was adapted to the optimal Chlamydomonas codon usage and interrupted by the first two *RBCS2* introns (Baier et al. 2018b; Schroda 2019). Moreover, the strong *HSP70A-RBCS2* promoter fusion (Schroda et al. 2000) and the strong *RPL23* terminator (Lopez-Paz et al. 2017) were used. Secretion of 2S albumin was mediated by the signal peptide from carbonic anhydrase (cCA) (Lauersen et al. 2013), which was most effective in mediating secretion of the SARS-CoV-2 spike protein (Kiefer et al. 2022). We assembled three transcription units for the production of 2S albumin with 8xHis at different positions (Fig. 1A). We combined the transcription units with the *aadA* cassette driven by the *PSAD* promoter and terminator and transformed them into Chlamydomonas. As recipient strain, we used UVM4 (Neupert et al. 2009) that had been transformed before with the *NIT1* and *NIT2* genes to enable growth of UVM4 on nitrate (N-UVM) (Freudenberg et al. 2021). Proteins in the culture medium of 12 spectinomycin-resistant transformants generated with each construct were precipitated with TCA and screened for the presence of secreted 2S albumin using an antibody against the HA epitope. Eight to eleven of the twelve transformants per construct were tested positive (Supplemental Fig. 1).

We then grew the best-expressing transformants for each construct in 100 ml TAP medium to stationary phase, when secretion of recombinant proteins is highest (Kiefer et al. 2022; Ramos-Martinez et al. 2017) and subjected the cell-free culture medium to Ni-NTA affinity chromatography. Recombinant 2S albumin was detected in the culture medium of all three transformants (InP in Fig. 1B). The 2S albumin variant with SP20-HA-8His was not depleted in the column flow-through and was absent in the eluates (FT, E1-4 in Fig. 1B, Supplemental Fig. 2), corroborating our results with the SARS-CoV-2 spike protein carrying this sequence (Kiefer et al. 2022) and indicating that the C-terminally exposed 8xHis tag is unsuitable for affinity purification. In contrast, 2S albumin with the 8His-SP20-HA and SP20-8His-HA sequences was depleted in the column flow-throughs and enriched in the eluates, indicating the suitability of the 8xHis tag for affinity purification if it is internalized.

**Fig. 2:**
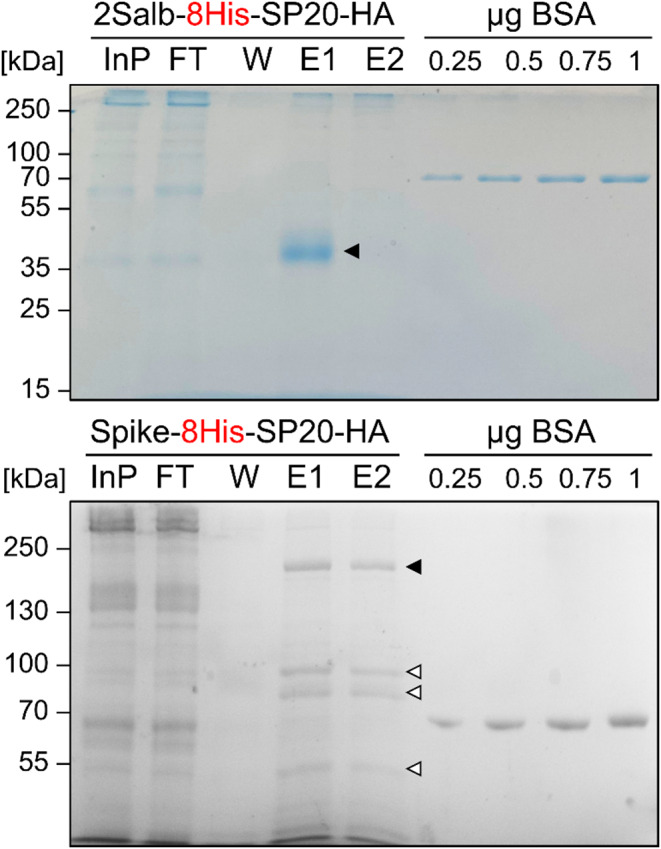
Purification of 2S albumin and SARS-CoV-2 spike protein ectodomain produced with internalized 8xHis tag. The two proteins were produced and secreted in 100 ml cultures of transformants #5 (2S albumin) and #17 (spike protein) (Supplemental Figs. 1 and 4) and purified from cell-free culture medium via Ni-NTA affinity chromatography. Proteins in 1 ml fractions from input (InP), flow-though (FT), and wash (W) as well as in 1.7 ml of the 2 ml eluates (E) were precipitated with TCA, separated on SDS-polyacrylamide gels and stained with Coomassie Blue. The indicated amounts of BSA were included for quantification. Full arrowheads point to the full-length proteins, open arrowheads point to degradation products. Shown are representative experiments, two more replicates for each protein are shown in Supplemental Fig. 3

To estimate the yield and purity of recombinant 2S albumin, we performed Ni-NTA affinity purification using 100 ml of culture medium from the 2S albumin variant containing the 8His-SP20-HA sequence. We then separated 85% of total eluted protein by SDS-PAGE and stained the gel with Coomassie Blue. As shown in Fig. 2 and Supplemental Fig. 3, the purified 2S albumin migrated with an apparent molecular mass of ~ 40 kDa, which is much larger than the calculated mass of 21.2 kDa after signal peptide cleavage. A larger apparent mass than the calculated mass was also observed for secreted Venus protein carrying the SP20 glycomodule, which was shown to be due to glycosylation (Ramos-Martinez et al. 2017). There were few impurities and no degradation products. Using densitometric quantification of the Coomassie-stained 2S albumin bands and BSA as a standard in three replicates, we estimated a yield of 31 ± 10.9 µg recombinant protein per liter of culture.

To test whether internalization of the 8xHis tag also enables the successful affinity purification of another recombinant protein, we assembled the coding sequence for the SARS-CoV-2 spike protein ectodomain (Kiefer et al. 2022) with the *HSP70A-RBCS2* promoter, the *RPL23* terminator, and sequences encoding the cCA signal peptide and the 8His-SP20-HA motifs into a vector containing the *aadA* cassette. Six out of 26 spectinomycin-resistant transformants produced and secreted detectable levels of the recombinant spike protein (Supplemental Fig. 4). Again, we grew the best-expressing transformant in 100 mL TAP medium to stationary phase and subjected the cell-free culture medium to Ni-NTA affinity chromatography. As shown in Fig. 2 and Supplemental Fig. 3, the internalized 8xHis tag in the 8His-SP20-HA sequence also enabled the successful purification of the spike protein. The protein migrated with an apparent molecular mass of ~ 200 kDa, which again is much larger than the calculated mass of 139 kDa, likely due to glycosylation of the SP20 glycomodule and the realization of at least some of the 22 N-linked glycans in the spike protein (Ramos-Martinez et al. 2017; Watanabe et al. 2020). As reported previously (Kiefer et al. 2022), the spike protein is unstable as evidenced by several degradation products, most prominently at ~ 96, ~85, and ~ 48 kDa. Densitometric estimation of the amount of intact spike protein and BSA as a standard yielded 23.3 ± 3.7 µg of recombinant spike protein per liter of culture.

## Discussion

We report here that the problem of unsuccessful affinity purification of secreted recombinant proteins from the culture medium of Chlamydomonas can be overcome by internalizing the affinity tag (Fig. 1). We demonstrate this with two different recombinant proteins: the methionine-rich 2S albumin from *Bertholletia excelsa* and the SARS-CoV-2 spike protein ectodomain (Fig. 2). We hypothesize that this problem is caused by proteolytic removal of the exposed tag, similar to what has been observed for secreted proteins in *Leishmania tarentulae* (Hieronimus et al. 2024). The efficient depletion of the tagged 2S albumin from the culture medium and the high purity of the recombinant protein after Ni-NTA affinity chromatography indicate that secreted recombinant proteins can efficiently be enriched when using an internalized 8xHis tag. This suggests that unassembled cell wall proteins that accumulate and aggregate in the extracellular space of Chlamydomonas cell wall deficient (*cw*) mutants and potentially trap secreted recombinant proteins (Baier et al. 2018a; Barolo et al. 2022), do not appear to pose problems. This is corroborated by the efficient purification also of the spike protein, although assessing its purity is more challenging due to its susceptibility to degradation (Fig. 2; Supplemental Figs. 3, 4) (Kiefer et al. 2022).

The successful purification of the two recombinant proteins to amounts that can be easily visualized by Coomassie Blue staining is promising. Nevertheless, a problem that remains to be solved is the low yield of recombinant protein of < 50 µg per liter of culture. For commercial purposes this needs to be boosted by at least ~ 100-fold. An ~ 10-fold improvement could be achieved by the cultivation of the transgenic lines in the N-UVM background in medium supporting high cell densities (Freudenberg et al. 2021). Further improvements could be achieved by enhancing secretion rates, for example, through the selection of more efficient signal peptides (Baier et al. 2018a; Molino et al. 2018), increasing protein folding capacity in the ER (Gasser et al. 2008), or translationally fusing the target protein with secretion-enhancing proteins such as the *Lolium perenne* ice-binding protein (Baier et al. 2018a; Lauersen et al. 2015). Additionally, fusing the target protein with easily detectable reporters, such as luciferase or fluorescent proteins, would facilitate screening for the highest-expressing transformants (Baier et al. 2018b; Lauersen et al. 2015). The degradation of unstructured C-termini in secreted recombinant proteins could represent a challenge when using Chlamydomonas as a platform for producing human therapeutic proteins, as it can lead to protein heterogeneity. A potential solution is the incorporation of cleavage sites for highly specific proteases, such as tobacco etch virus (TEV) protease or PreScission protease, upstream of the affinity tag. This would allow for precise tag removal, as well as the elimination of other undesired sequences such as the SP20 glycomodule, stabilizing proteins, or reporters, upon elution of the target protein from the affinity matrix. While challenges particular regarding the yield of recombinant proteins still need to be addressed, this study represents an important step toward establishing Chlamydomonas as a viable and sustainable platform for the production of high-value recombinant proteins.

## Methods

### Strains and culture conditions

*Chlamydomonas reinhardtii* strain UVM4 (Neupert et al. 2009) was transformed with plasmids containing the wild-type *NIT1* (pMN24, Fernandez et al. (1989) and *NIT2* (pMN68, Schnell and Lefebvre (1993) genes via the glass beads method (Kindle 1990). Transformants were selected on Tris-Acetate-Phosphate (TAP)-NO_3_ agar plates (Kropat et al. 2011). A transformant able to grow on nitrate-containing medium (N-UVM) was then transformed with constructs pMBS1119, pMBS607, pMBS1047, and pMBS1026 (see below for construction details) linearized with *Not*I. Transformants were selected on TAP-agar plates containing 100 µg mL^− 1^ spectinomycin (Merck). For secretion assays, transformants were grown mixotrophically in TAP-NH_4_-medium at 25 °C at 120 rpm orbital shaking under continuous light of ~ 70 µmol photons m^− 2^ s^− 1^.

### Plasmid design and cloning

The amino acid sequence of *Bertholletia excelsa* 2S albumin (Uniprot accession B6EU54) was reverse translated using the optimal *Chlamydomonas* codon usage. The first two Chlamydomonas *RBCS2* introns were inserted with the flanking site AG/intron/GA (Baier et al. 2018b). The coding sequence was flanked by *Bbs*I recognition sites and the overhangs AATG and TTCG for position B3–B4 of level 0 parts according to the MoClo standard for Chlamydomonas (Crozet et al. 2018). Gene synthesis and cloning into pUC57 was done by TWIST resulting in pMBS483. To swap the position of the 8xHis tag around the SP20 glycomodule, PCRs were performed on Level 0 construct pMBS659 (SP20-HA-8xHis) (Kiefer et al. 2022) using primers 5’-AAGAAGACAATTCGCACCACCACCACCACCACCACCACTCGCCCTCGCCCAGCCC-3’ and 5’-TTGAAGACAAAAGCTTAAGCGTAGTCGGGCACGT-3’, and 5’-AAGAAGACAACCACGgcagctacccttacgacgtgccggactacgcctaaGCTTTGAGACCTTATCG-3’ and 5’-AAGAAGACTAGTGGTGGTGATGGTGGT-3’ to generate genetic parts encoding 8His-SP20-HA (pMBS1022) and SP20-8His-HA (pMBS818), respectively. Each PCR product and pAGM1301 (Weber et al. 2011) were digested with *Bbs*I (NEB) and ligated with T4 DNA ligase (NEB) in a one-pot reaction, resulting in pMBS1022 and pMBS818, respectively. For the production and secretion of 2S albumin and spike protein, their encoding level 0 parts B3-B4-pMBS483 [2S albumin] and B3-pMBS706 [CoV2-S up] / B4-pMBS708 [CoV2-SGSAS/PP-down] were assembled with A1-B1-pCM0-015 [*HSP70A-RBCS2* promoter + 5′-UTR], B2-pCM0-051 (cCA signal peptide), B5-pMBS659 [SP20-HA-8His] / B5-pMBS1022 [8His-SP20-HA] / B5-pMBS818 [SP20-8His-HA], and B6-C1-pCM0-119 (*RPL23* 3′UTR terminator) (Crozet et al. 2018; Kiefer et al. 2022) into the level 2 destination vector pMBS807 conferring resistance to spectinomycin (Niemeyer and Schroda 2022) in a one-pot reaction with *Bsa*I (NEB) and T4 DNA ligase (NEB) yielding vectors pMBS1119 (Spike-8His-SP20-HA), pMBS1047 (2Salb-SP20-8His-HA), and pMBS1026 (2Salb-8His-SP20-HA) (see Schroda and Remacle (2022) for an overview of the assembly strategy). In case of the construct producing 2S-albumin fused to the SP20-HA-8His sequence, the parts were first assembled into Level 1 recipient vector pICH47742 yielding pMBS606, which was then combined with pCM1-01 (level 1 module with the *aadA* cassette flanked by the *PSAD* promoter and terminator) with plasmid pICH41744 containing the proper end-linker, and with destination vector pAGM4673, giving pMBS607. All constructs were verified by Sanger sequencing.

### SDS-PAGE, immunostaining and coomassie blue staining

TCA precipitation and sample preparation of secreted proteins for SDS-PAGE were performed as previously described in Kiefer et al. (2022). Samples with 2S albumin were analysed on 12% polyacrylamide gels while those with spike protein on 8% polyacrylamide gels. After gel electrophoresis, gels were either stained with colloidal Coomassie Blue or transferred to a nitrocellulose membrane using semidry blotting and immunostained with anti-HA (H9658, Merck, 1:10,000). m-IgGκ BP-HRP (sc-516102, Santa Cruz Biotechnology, 1:10,000) was used as secondary antibody. Chemiluminescence signals were visualized using an INTAS imaging system. Coomassie-stained bands of 2S albumin, the spike protein, and the BSA standard were quantified densitometrically using ImageJ. The amounts of recombinant proteins were estimated through linear regression based on the BSA standard. Finally, mean values and standard deviations were calculated from three independent replicates.

### Purification of secreted proteins by Ni-NTA affinity chromatography

Cultures were initially diluted to a cell density of 1*10^5^ cells/mL and then grown to stationary phase for six days. 100 mL growth medium was separated from cells by centrifuging twice for 2 min at 4,000 g and 25 °C. After addition of NaCl to a final concentration of 300 mM, the cell-free medium was loaded onto a column containing 1 mL of Ni-NTA (P6611, Merck) slushy equilibrated with one column volume column buffer (300 mM NaCl, 20 mM TRIS pH 8.0). Afterwards the column was washed with 10 mL column buffer containing 5 mM imidazole. Proteins were eluted with 4 mL 500 mM imidazole in two fractions of 2 mL each.

## Electronic supplementary material

Below is the link to the electronic supplementary material.

Supplementary Material 1

## Data Availability

No datasets were generated or analysed during the current study.
