# Supplementary material for: Internalization of affinity tags enables the purification of secreted Chlamydomonas proteins
Source: Curr Genet. 2025 Mar 19;71(1):7. doi: 10.1007/s00294-025-01311-2 (PMC11923035; doi:10.1007/s00294-025-01311-2)
Supplement: Supplementary file 1 — Supplementary Material 1 [file 294_2025_1311_MOESM1_ESM.docx]

**
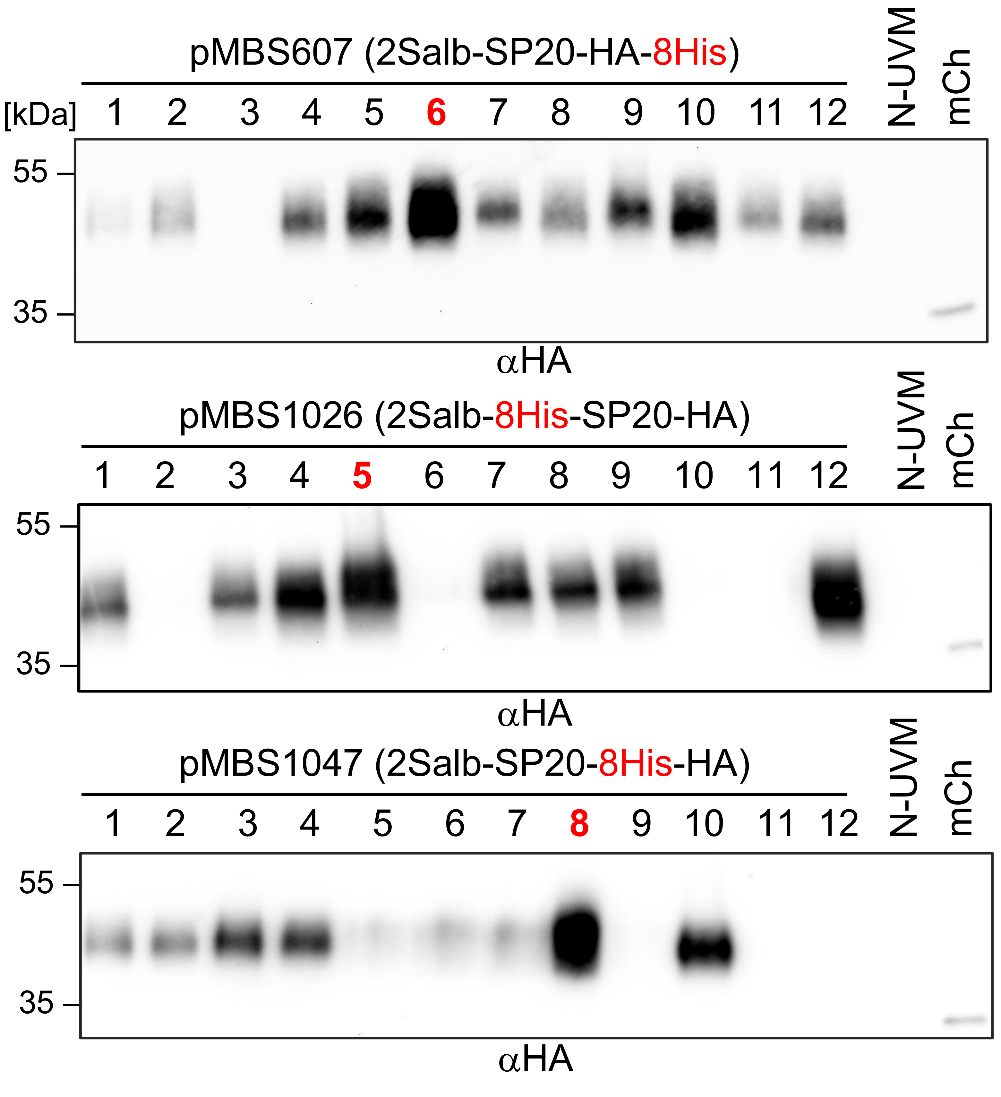
**

**Supplemental Fig. 1** Generation of Chlamydomonas lines producing and secreting 2S albumin. Chlamydomonas strain N-UVM was transformed with the indicated level 2 constructs depicted in Fig. 1A. Twelve spectinomycin-resistant transformants for each construct were grown in TAP medium. Proteins in 1.7 ml of culture medium for each transformant and N-UVM as negative control were precipitated with TCA and analyzed by SDS-PAGE and immunoblotting using an antibody against the HA epitope. Transformants selected for further analyses are highlighted in red. 10 ng recombinant mCherry-6His-HA (mCh) from Kiefer et al. (2022) were loaded as positive control.

**
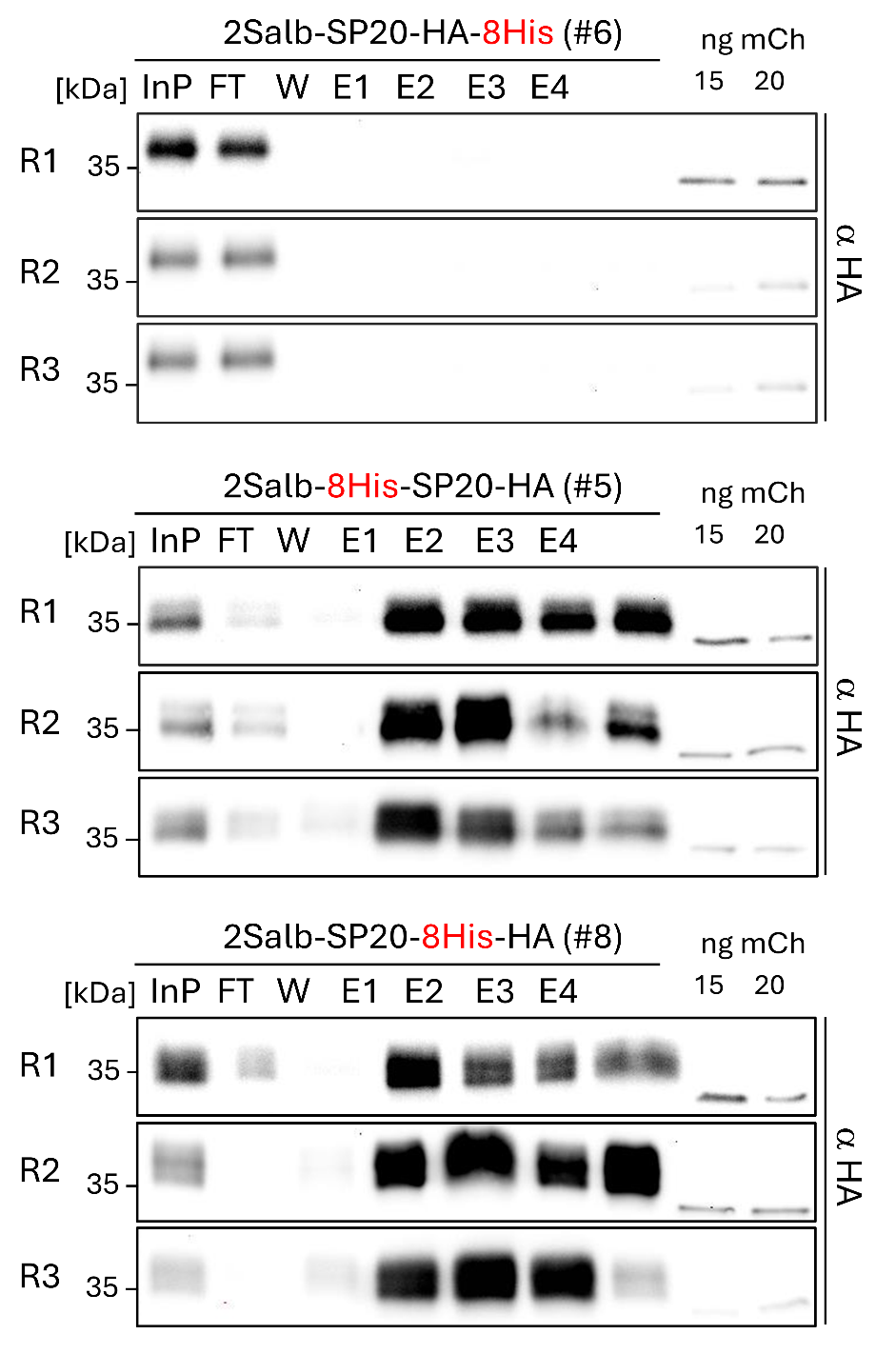
**

**Supplemental Fig. 2** All replicates (R1-3) for the purification of 2S albumin harboring the 8xHis tag at different positions. See legend of Fig. 1 for details.

**
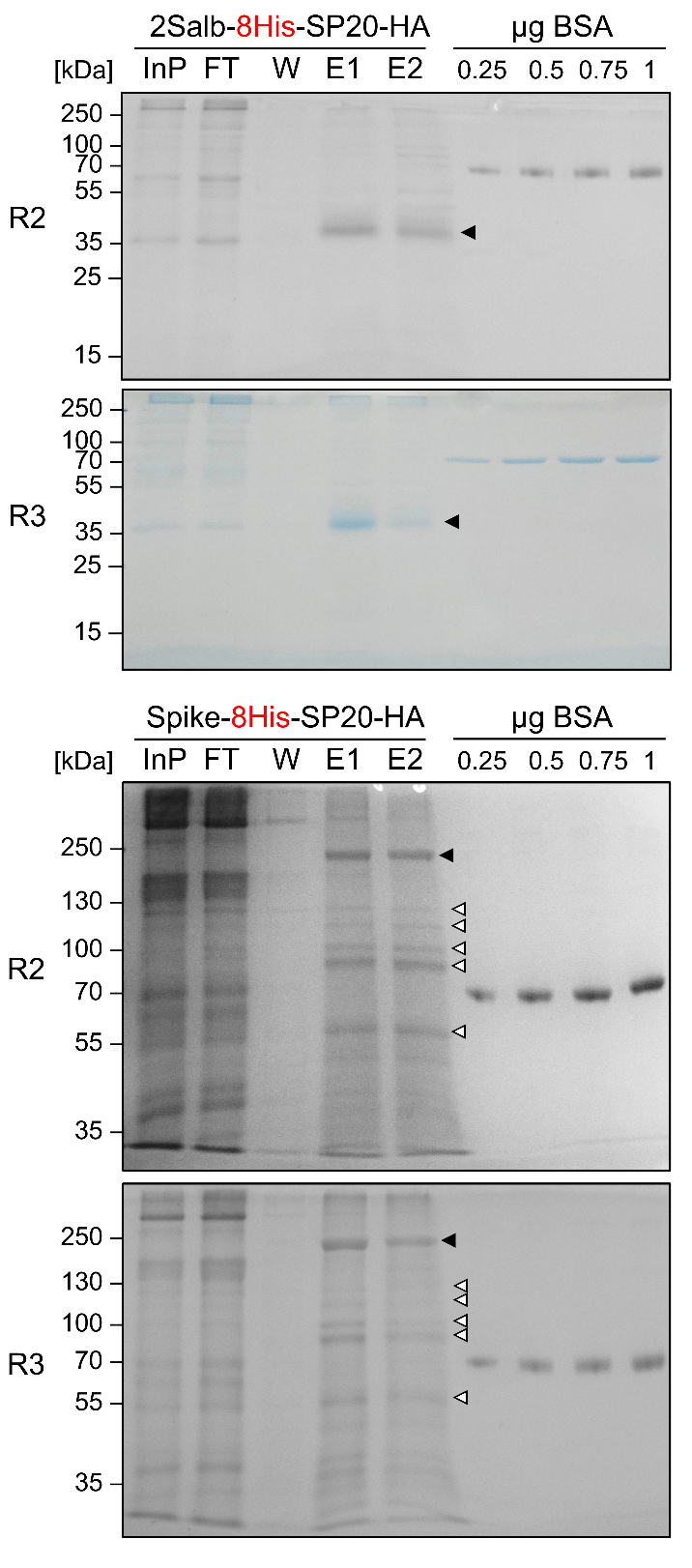
**

**Supplemental Fig. 3** Two more replicates (R2-3) for the purification of 2S albumin and the SARS-CoV-2 spike protein ectodomain harboring an internalized 8xHis tag. See legend of Fig. 2 for details.

**
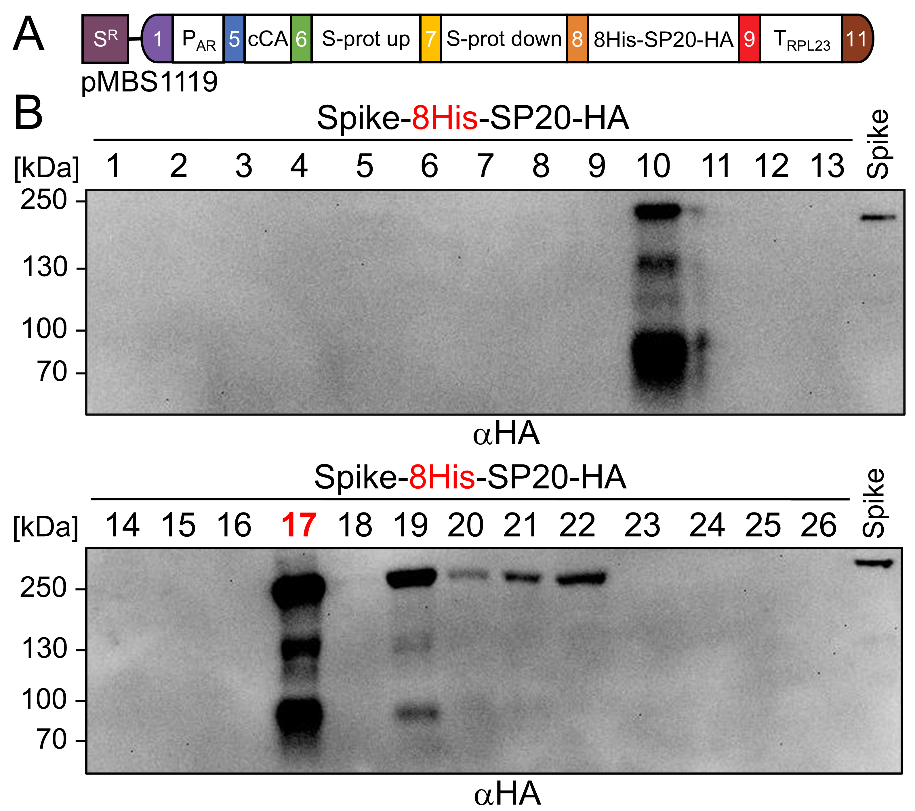
**

**Supplemental Fig. 4** Generation of Chlamydomonas lines producing and secreting the SARS CoV-2 spike protein. **A** MoClo level 2 construct pMBS1119 for the production of the SARS CoV-2 spike protein in the prefusion-stabilized conformation and without the membrane anchor and the furin cleavage site (Kiefer et al. 2022) with internalized 8xHis affinity tag. The coding sequence for the spike protein was split in two parts (up/down), the other parts employed are described in Fig.1. **B** Screening for transformants secreting the spike protein. pMBS1119 was transformed into Chlamydomonas strain N-UVM. 26 spectinomycin-resistant transformants were grown in TAP medium. Proteins in 1.7 ml of culture medium for each transformant were precipitated with TCA and analyzed by SDS-PAGE and immunoblotting using an antibody against the HA epitope. The transformant selected for further analyses is highlighted in red. Spike protein with SP20-HA-8His directly precipitated from the culture medium (Kiefer et al. 2022) was loaded as positive control (Spike).
